# Supplementary material for: Classifying cold‐stress responses of inbred maize seedlings using RGB imaging
Source: Plant Direct. 2019 Jan 2;3(1):e00104. doi: 10.1002/pld3.104 (PMC6508840; doi:10.1002/pld3.104)
Supplement: Supplementary file 1 [file PLD3-3-e00104-s001.pdf]

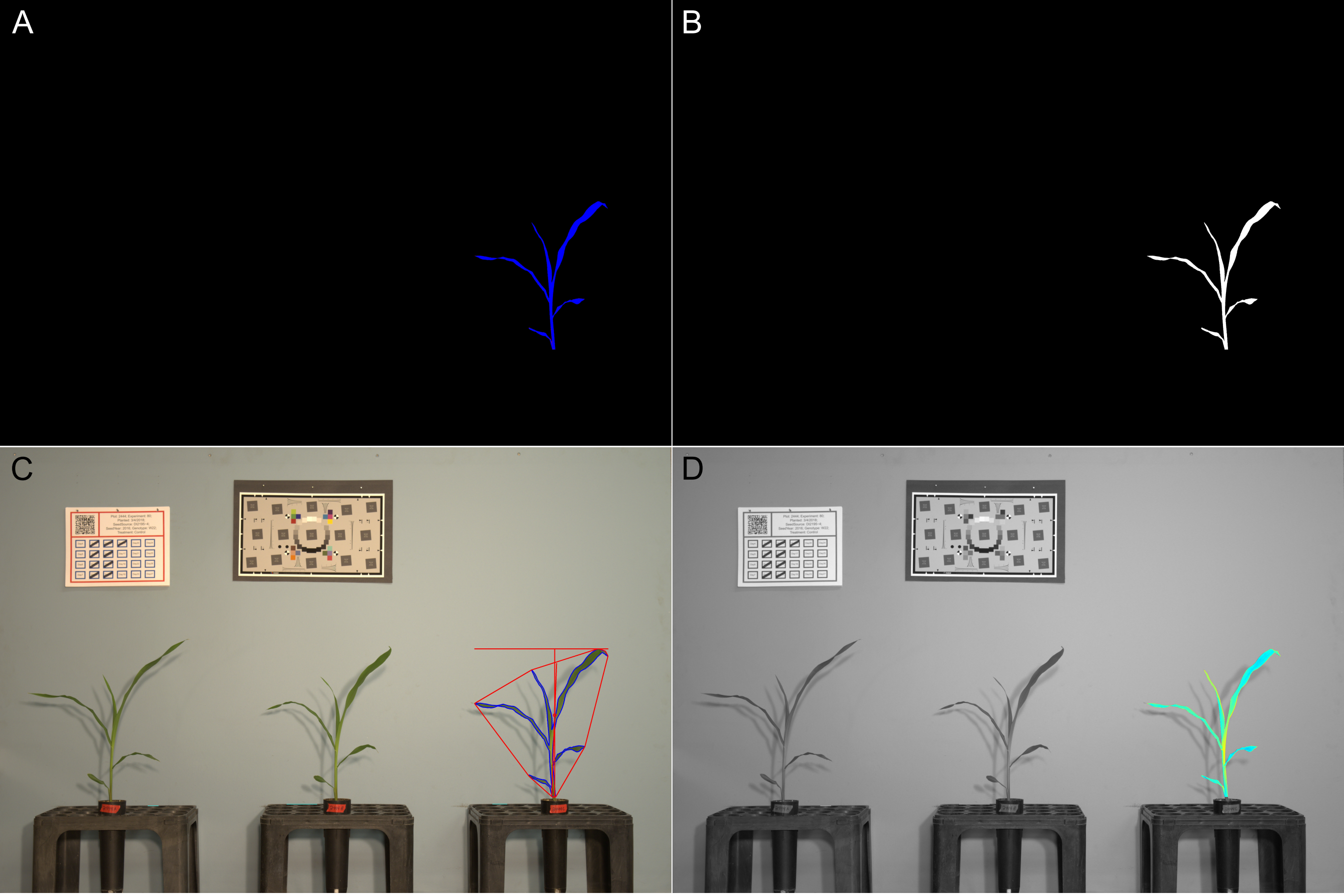

Supplemental Figure 1. Image output from PlantCV pipeline. A) Merged image indicates pixels categorized as healthy (blue) or necrotic (red). B) Final plant binary mask used for shape and color analysis. C) Output from PlantCV analyze_object function. D) Output from PlantCV analyze_color function false colored using the "value" channel from HSV colorspace.
