# Supplementary figures and images for: Classifying cold‐stress responses of inbred maize seedlings using RGB imaging
Source: Plant Direct. 2019 Jan 2;3(1):e00104. doi: 10.1002/pld3.104 (PMC6508840; doi:10.1002/pld3.104)

area

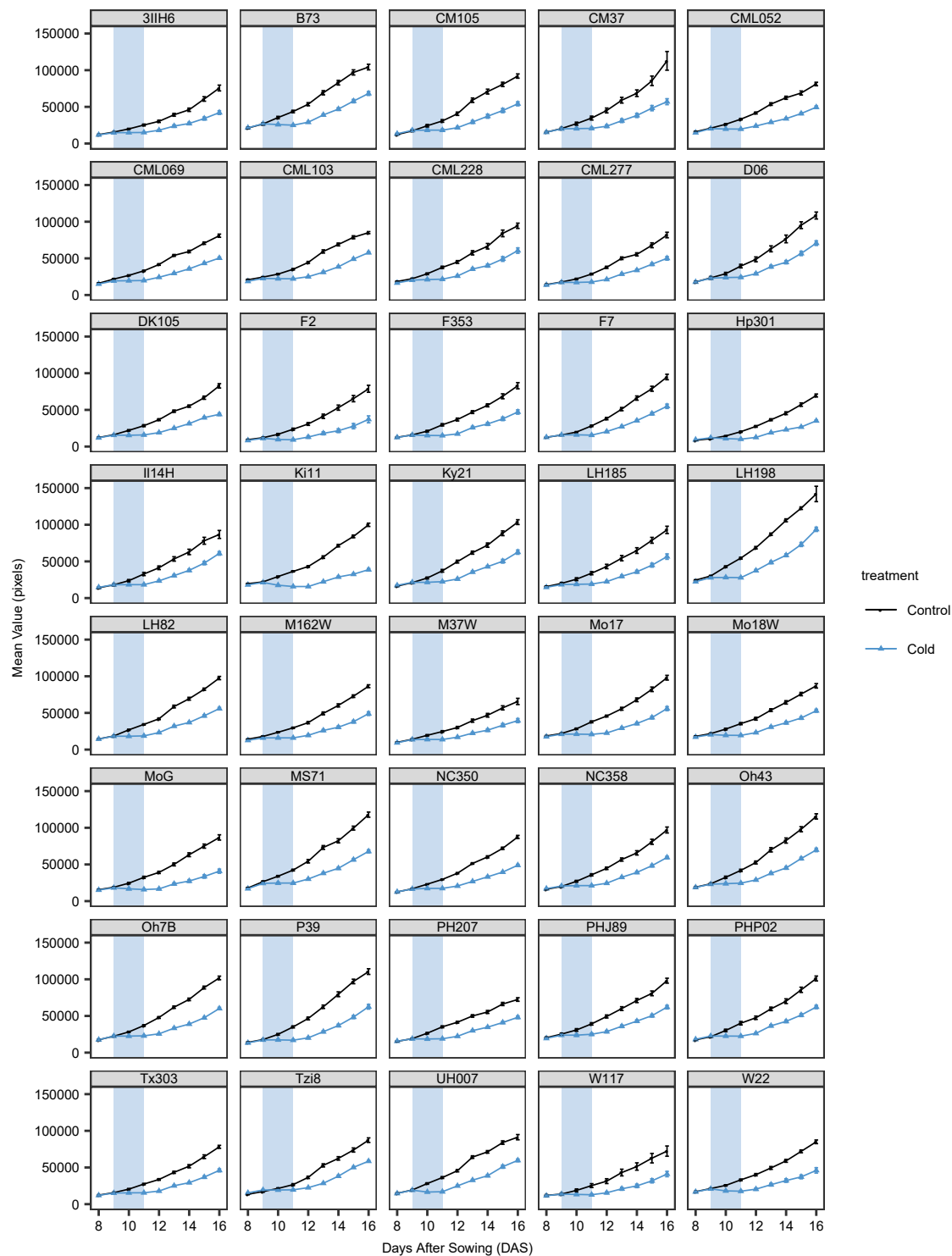

Supplement: Supplementary file 3 [file PLD3-3-e00104-s003.pdf]
