# Supplementary material for: Classifying cold‐stress responses of inbred maize seedlings using RGB imaging
Source: Plant Direct. 2019 Jan 2;3(1):e00104. doi: 10.1002/pld3.104 (PMC6508840; doi:10.1002/pld3.104)
Supplement: Supplementary file 5 [file PLD3-3-e00104-s005.pdf]

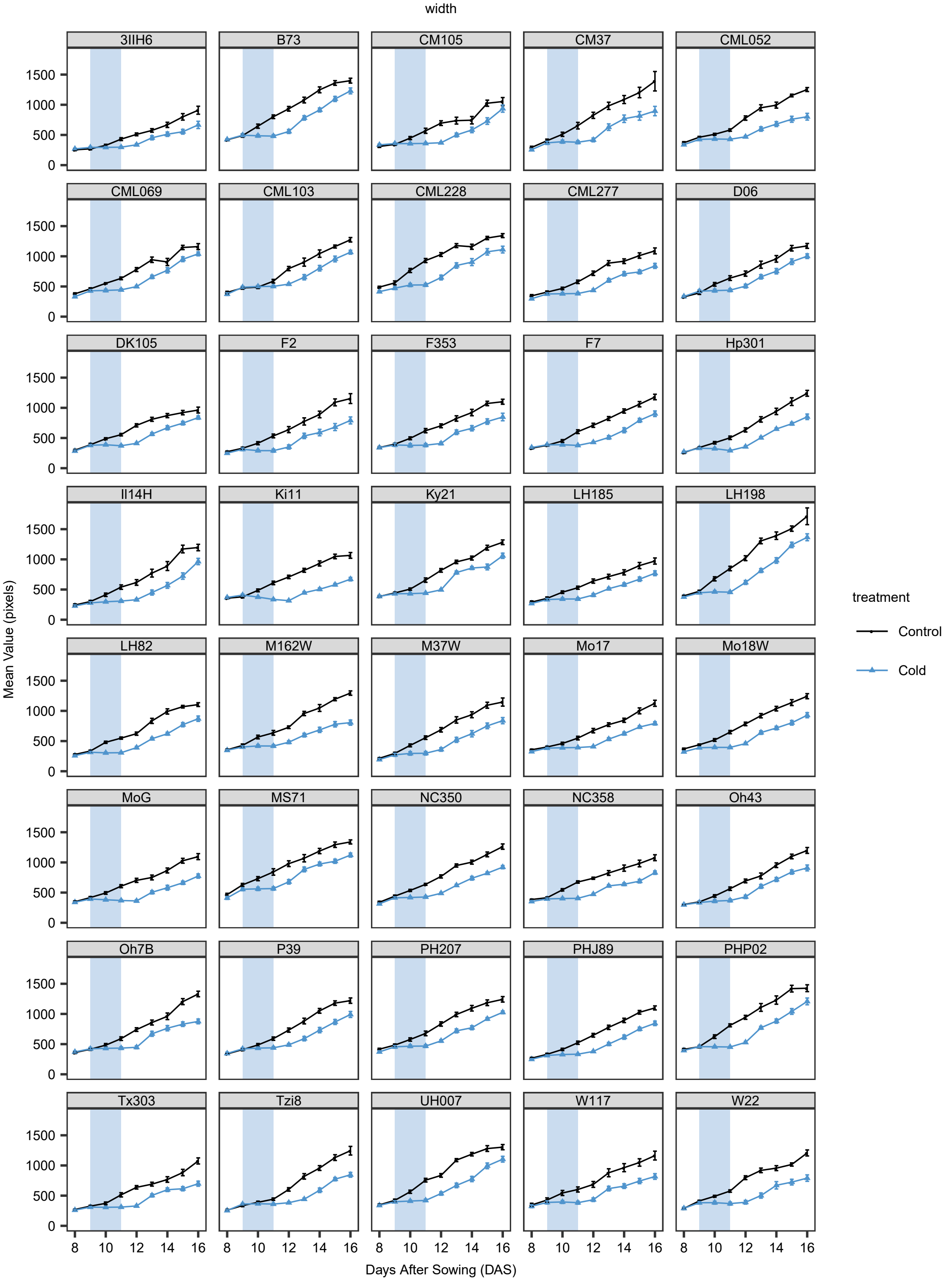

Supplemental Figure 5. Mean values for plant width at each timepoint for 40 maize inbred genotypes. Error bars represent standard error of the mean.
