# Supplementary material for: Classifying cold‐stress responses of inbred maize seedlings using RGB imaging
Source: Plant Direct. 2019 Jan 2;3(1):e00104. doi: 10.1002/pld3.104 (PMC6508840; doi:10.1002/pld3.104)
Supplement: Supplementary file 8 [file PLD3-3-e00104-s008.pdf]

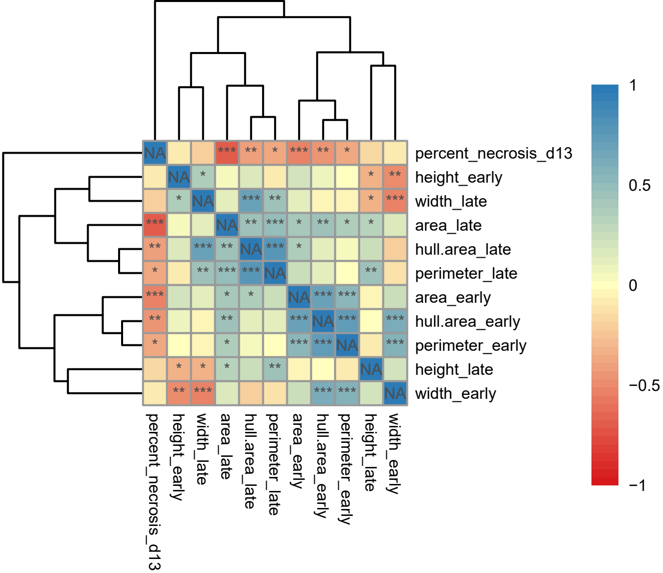

Supplemental Figure 8. Correlations between traits. Correlation matrix of log2 fold-change values of growth rates between treatment groups for all genotypes for each trait within each interval. Values represent Pearson's r value. Asterisks indicate significance level of P value (* P ≤ 0.05; ** P ≤ 0.01; *** P ≤ 0.001).
