# Supplementary material for: Classifying cold‐stress responses of inbred maize seedlings using RGB imaging
Source: Plant Direct. 2019 Jan 2;3(1):e00104. doi: 10.1002/pld3.104 (PMC6508840; doi:10.1002/pld3.104)
Supplement: Supplementary file 11 [file PLD3-3-e00104-s011.pdf]

Editors comments:

In order to insure a full reproducibility and durability of the shared codes, could you create a DOI for your github repository (https://github.com/maizeumn/cold-phenotyping), for instance Zenodo (https://guides.github.com/activities/citable-code/). this will create a stable, long term archival of the code used for this manuscript (which github alone does not provide).

Thank you for this suggestion. We have created a DOI for our github repository here: http://doi.org/10.5281/zenodo.1553411 and included this link as appropriate in the text.

Reviewer #1:

I have only one problem with the analysis of the data which relates to the clustering. Clearly the amplitude of differences is very different between traits and scaled to the strongest response (the percent necrosis on day 13 and height early recovery). This means that the differences in the other traits are marginal and presumably do not contribute much to the clustering. In analogy to clustering of transcriptome data, it should be tried to normalise the "trait" data (so they all have the same amplitude) prior to clustering. This may or may not alter the relationship with genetic or geographic origin, but should in any case be tried.

It is correct that the clustering is largely driven by these two traits, but the other traits do contribute to the clustering. We addressed the clustering clearly depends on which traits were chosen and how intervals were defined in our discussion on lines 377-381.

We also normalized the $\log_2$(FC) growth rate values within each interval for each trait (see figure below). We concluded that although there are small differences between which genotypes fall into different clusters, our overall conclusions stated regarding the genetic and geographic origin and their relationship to cold tolerance have not changed. Therefore, we decided to keep the original heatmap as Figure 7.

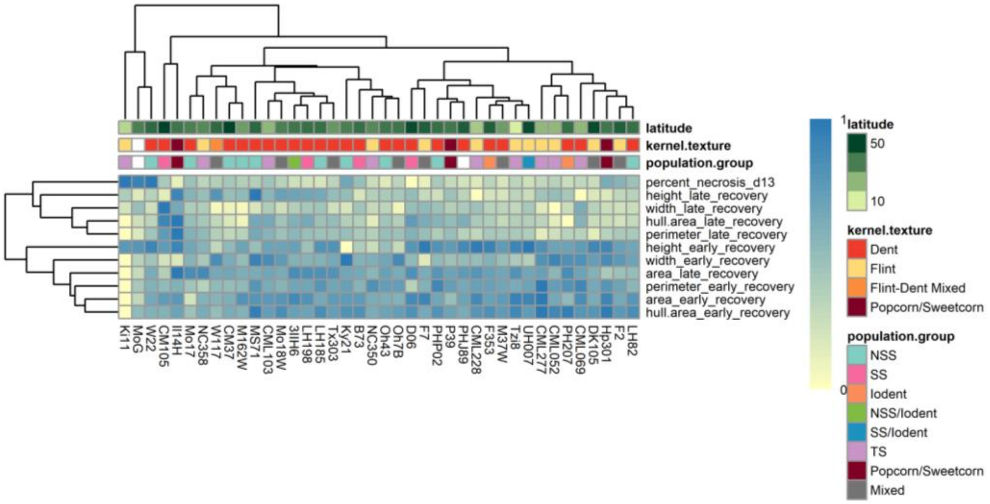

Moreover, in parallel to clustering of the genotypes, clustering of the traits should be performed to see how these traits relate to each other. This would give more insight into the recovery response of the different groups of genotypes.

We have edited the heatmap in Figure 7 to cluster rows as well as columns, adding some information on how traits were related. In addition, we have created a correlation matrix for the

values used in the heatmap in Figure 7 to further investigate how traits were related (see Supplemental Figure 8). We have described this data on lines 337-344.

Reviewer #2:

Lines 77-79: As "staging" can mean either determining the growth stage of a plant, or placing the plants in the location where they will be imaged, and both are potentially applicable here, is there another term you could use? (Optional)

Thank you, we agree this word choice could be improved. We have changed “staging” to “placement” on line 78.

Lines 95-96: Was the Bayesian classifier from PlantCV used in this paper? I cannot find any mention of it in methods. If not, perhaps omit this line as it seems possible it would confuse readers.

Yes, we did use the Bayesian classifier from PlantCV to quantify the percent plant area classified as healthy or necrotic tissue. To make this clearer, we have added the phrase “using the Naïve Bayes classifier” to the end of the next sentence on lines 97-100. Please see lines 170-174 describing the use of the Naïve Bayes Classifier in our methods section.

Lines 128-134: would it be possible to either deposit these scripts online or include them as supplemental material?

These scripts are available in our github repository mentioned throughout our methods section, and located at https://github.com/maizeumn/cold-phenotyping (doi: 10.5281/zenodo.1553411). To make this more explicit, we have added a sentence stating that these scripts are available at the specified link on lines 134-136.

Line 375-376: Since Ki11 was developed in Thailand by Kasetsart University, this doesn't seem likely to be a great representative of flint-type maize lines as a whole and the tropical lattitude origin should probably be mentioned if Ki11 is highlighted.

We have added a statement drawing attention that Ki11 is of tropical origin on line 391.

Lines 383-393: It might help to include a little discussion in this paragraph about how this is an issue for comparatively recently developed maize lines, but not for maize landraces, which show significant adaption to the specific environments in which they were collected. See Romero Novarro et al doi: 10.1038/ng.3784

We have added a sentence on lines 398-400 discussing this point and added this citation. Thank you for this feedback.
